# Intrinsically altered lung‐resident γδT cells control lung melanoma by producing interleukin‐17A in the elderly

**DOI:** 10.1111/acel.13099

**Published:** 2020-01-05

**Authors:** Min Cheng, Yongyan Chen, Dake Huang, Wen Chen, Weiping Xu, Yin Chen, Guodong Shen, Tingjuan Xu, Gan Shen, Zhigang Tian, Shilian Hu

**Affiliations:** ^1^ Gerontology Institute of Anhui Province The First Affiliated Hospital of University of Science and Technology of China (Anhui Provincial Hospital) Hefei China; ^2^ Anhui Provincial Key Laboratory of Tumor Immunotherapy and Nutrition Therapy Hefei China; ^3^ Cancer Immunotherapy Center The First Affiliated Hospital of University of Science and Technology of China (Anhui Provincial Hospital) Hefei China; ^4^ Institute of Immunology and The CAS Key Laboratory of Innate Immunity and Chronic Disease School of Life Science and Medical Center University of Science and Technology of China Hefei China; ^5^ Comprehensive Laboratory School of Basic Medical Sciences Anhui Medical University Hefei China

**Keywords:** aging, commensal microbiota, interleukin‐17A, lung cancer, lung‐resident γδT cell

## Abstract

Cancer is an age‐associated disease, potentially related to the altered immune system of elderly individuals. However, cancer has gradually decreased incidence in the eldest globally such as the most common lung cancer, the mechanisms of which remain to be elucidated. In this study, it was found that the number of lung‐resident γδT cells was significantly increased with altered gene expression in aged mice (20–24 months) versus young mice (10–16 weeks). Aged lung Vγ4^+^ and Vγ6^+^ γδT cells predominantly produced interleukin‐17A (IL‐17A), resulting in increased levels in the serum and lungs. Moreover, the aged mice exhibited smaller tumors and reduced numbers of tumor foci in the lungs after challenge with intravenous injection of B16/F10 melanoma cells compared with the young mice. Aged lung Vγ4^+^ and Vγ6^+^ γδT cells were highly cytotoxic to B16/F10 melanoma cells with higher expression levels of CD103. The markedly longer survival of the challenged aged mice was dependent on γδT17 cells, since neutralization of IL‐17A or depletion of indicated γδT cells significantly shortened the survival time. Consistently, supplementation of IL‐17A significantly enhanced the survival time of young mice with lung melanoma. Furthermore, the anti‐tumor activity of aged lung γδT17 cells was not affected by alterations in the load and composition of commensal microbiota, as demonstrated through co‐housing of the aged and young mice. Intrinsically altered lung γδT17 cells underlying age‐dependent changes control lung melanoma, which will help to better understand the lung cancer progression in the elderly and the potential use of γδT17 cells in anti‐tumor immunotherapy.

## Graphical abstract

In the aged mice (20–24 months), the number of lung‐resident γδT cells was significantly increased with altered gene expression compared with the young mice (10–16 weeks). Aged lung γδT cells predominantly produced interleukin‐17A (IL‐17A), which played a critical role in resistance to the development of lung melanoma in the aged. Furthermore, the anti‐tumor activity of aged lung γδT17 cells was not affected by alterations in the load and composition of commensal microbiota.

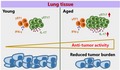

## 1 INTRODUCTION

Similar to other tissues, the immune system changes with age, which has been recognized based on the increased incidence and mortality rate of infectious disease and a wide range of age‐associated diseases in the elderly. In particular, cancer is one of these age‐associated diseases, with the age‐specific incidence rate reaching its peak at the age group of 85–89 years in UK (Data from CRUK) and 80–84 years in China (Chen et al., 2017). Lung cancer is the most commonly occurring type of cancer (11.6% of the total cases globally; 17.08% of the total cases in China) and the leading cause of cancer death (18.4% of the total cancer deaths globally; 21.68% of the total cancer deaths in China; Bray et al., 2018; Chen, Zheng, et al., 2016a). The incidence rate of lung cancer significantly changes with age. In United States, the incidence rate of lung cancer peaked at the age of 80–84 years in males and 75–79 years in females and then gradually decreased (Dela Cruz, Tanoue, & Matthay, 2011); in UK, the incidence rate of lung cancer peaked at the age of 85–89 years in males and 80–85 years in females (Data from CRUK); and in China, the incidence of lung cancer peaked at the age group of 60–74 years both in males and females, and then gradually decreased in individuals over 75 years old, and also for the mortality rate (Chen, Zheng, et al., 2016a). Notably, advanced lung cancer associated with a worse prognosis is more frequently reported in younger versus older patients and the underlying mechanism is currently unknown (Bourke et al., 1992; Chen, Lai, et al., 2016b). Studies using a murine B16 lung melanoma metastasis model demonstrated fewer pulmonary colonies and slower growth in the lungs and longer survival in aged mice (Chen et al., 2007; Ershler, Stewart, Hacker, Moore, & Tindle, 1984). The incidence and progression of tumors in the aged lungs are worthy of further investigation.

It is well known that immune surveillance is important to prevent the development of cancer. However, a hallmark of aging is the progressive dysfunction of the immune system (Raynor, Lages, Shehata, Hildeman, & Chougnet, 2012). Age is associated with the reduced development and impaired function of CD4^+^T, CD8^+^T, and B cells, and decreased cellular function of innate immune cells including neutrophils, macrophages, monocytes, dendritic cells, and natural killer (NK) cells (Nikolich‐Zugich, 2018; Shaw, Goldstein, & Montgomery, 2013; Solana et al., 2012). On the other hand, in aged mice (generally >20 months) and humans (>65 years), activation of the innate immune system results in dysregulated inflammation. These populations are in a basal systemic inflammatory state termed “inflammaging,” characterized by high levels of pro‐inflammatory cytokines such as interleukin‐1 (IL‐1), IL‐6, IL‐8, tumor necrosis factor (TNF)‐α, and C‐reactive protein (Shaw et al., 2013). These alterations in the immune system resulted in impairment of efficient innate and adaptive immune responses to new pathogens or antigens, potentially contributing to age‐associated diseases in elderly individuals (Franceschi & Campisi, 2014; Shaw et al., 2013). In aged lungs, increased levels of TNF‐α, IL‐6, surfactant proteins and lipids (e.g., SP‐A and SP‐D), and several complement components were observed (Moliva et al., 2014). Additionally, in both animals and humans, aging is associated with increased production of IL‐10, which suppresses innate pulmonary granuloma cytokine response, innate interferon (IFN)‐γ production, and consequently Th1 cell priming (Chiu, Stolberg, & Chensue, 2008; Chiu, Stolberg, Freeman, & Chensue, 2007). Thus, the age‐related immune responses in the lung remain to be fully elucidated.

Abundant γδT cells—accounting for approximately 8%–20% of resident pulmonary lymphocytes in the lung—play critical roles as a bridge between the innate and adaptive immune systems for maintaining lung immune homeostasis (Cheng & Hu, 2017). However, thus far, few studies have investigated the immune functions of aged γδT cells. In fact, the pattern of Vγ gene usage of lung‐resident γδT cells changes with age. Vγ6^+^ γδT cells are the major γδT‐cell population from birth to 8–10 weeks of age, whereas Vγ4^+^ γδT cells predominate in older ages (Sim, Rajaserkar, Dessing, & Augustin, 1994). In normal adult C57BL/6 mice, a population of 2–5 × 10^4^ γδT cells is divided into subsets expressing Vγ4^+^ (~45%), Vγ1^+^ (~15%), Vγ6^+^ (~20%), and Vγ7^+^ (rare); Vγ5^+^ is absent (Sim et al., 1994; Wands et al., 2005). Functionally, γδT cells are classified into two subsets: IFN‐γ‐producing γδT cells (γδT1) and IL‐17‐producing γδT cells (γδT17). γδT17 cells develop and differentiate in embryonic thymus, and subsequently persist in adult mice as self‐renewing, long‐lived cells (Chien, Zeng, & Prinz, 2013; Haas et al., 2012); however, evidence of lymphoid precursors in the lungs indicates that γδT cells may undergo differentiation and selection in the lung (Sim et al., 1994). γδT17 cells depend upon transforming growth factor (TGF)‐β, rather than IL‐23 or IL‐6 for their development and maintenance; however, they are activated by IL‐1β plus IL‐23 (Ma et al., 2011). The population generated in situ and not selected in the thymus may include the γδT cells that are typical for the pulmonary environment (Sim et al., 1994). Additionally, there is another set of γδT17 cells referred to as the “inducible” γδT17 cells, which mature and differentiate to produce IL‐17 after antigen encounter in the secondary lymphoid organs (Chien et al., 2013). Therefore, it is necessary to study lung‐resident γδT cells in the elderly.

In this study, we identified lung‐resident γδT cells in the aged mice compared with the young. Poorly immunogenic B16/F10 melanoma that colonizes to the lung was used to study their anti‐tumor activity in the lung as previously reported (Martin‐Orozco et al., 2009). Intrinsically altered lung γδT cells predominantly produced IL‐17A and played a critical role in resistance to the development of lung melanoma in the aged. The findings will help to better understand the lung cancer progression in the elderly.

## 2 RESULTS

### 2.1 Increased number of lung‐resident γδT cells with altered gene expression in aged mice

**Figure 1:**
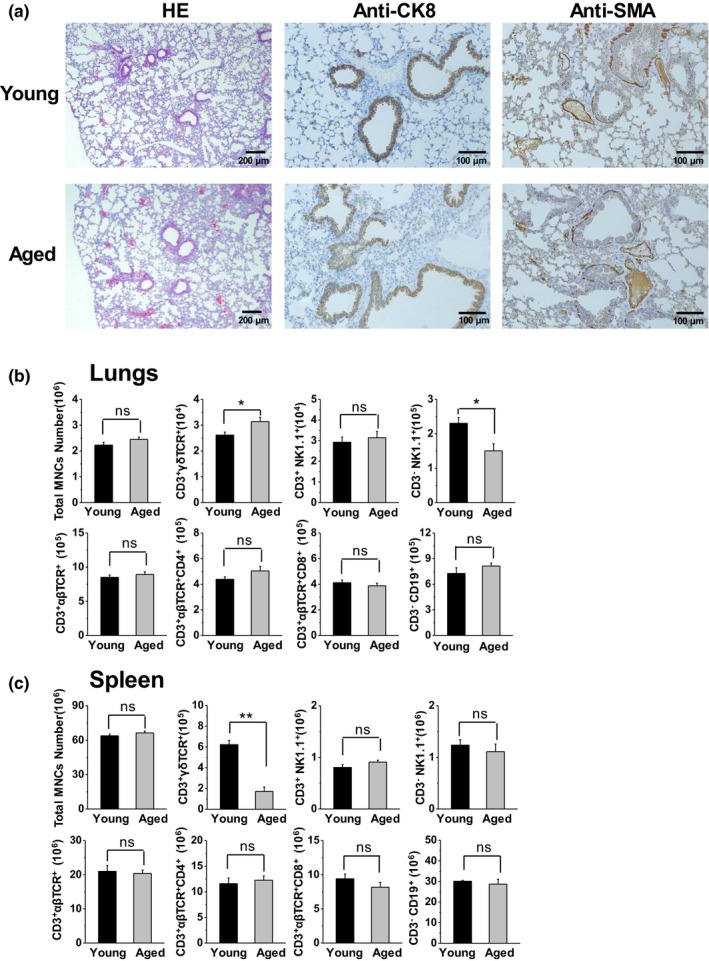
Increased number of γδT cells in the lungs of aged mice compared with young mice. (a) Lung samples were collected from young mice (10–16 weeks) and aged mice (20–24 months) for hematoxylin and eosin staining, and histochemical analysis using anti‐α‐SMA and anti‐CK8 antibodies. (b&c) The MNCs were isolated and analyzed using FACS. Lymphocytes were gated through FSC and SSC. The total number of MNCs and the absolute number of each lymphocyte subset in the lungs (b) and spleen (c) are shown (*n* = 6). The data are shown as the mean ± *SEM*. Student's *t* test was used. *p < .05, **p < .01. CK8, cytokeratin 8; FACS, fluorescence‐activated cell sorting; FSC, forward scatter; MNCs, mononuclear cells; *SEM*, standard error of the mean; SSC, side scatter; α‐SMA, alpha‐smooth muscle actin

The aged lung loses alveolar complexity and surface area (Mauderly & Hahn, 1982). We determined the morphological structure of lung tissue in the aged mice. The morphology was consistent with that reported when compared to the young mice as shown by the hematoxylin–eosin (HE) staining (Figure 1a). There were no significant differences observed in the epithelial hyperplasia and the lung vascular walls between the aged and young mice, confirmed by the expression of cytokeratin 8 (CK8) and alpha‐smooth muscle actin (α‐SMA) in the lung tissue, respectively (Figure 1a). There was no difference in the total number of mononuclear cells (MNCs) in the lungs of aged mice compared with young mice (Figure 1b). However, the number of γδT cells was significantly increased, whereas the number of NK cells was decreased (Figure 1b). There was no significant difference in the number of NKT, CD4^+^T, and CD8^+^T cells in the lungs between the aged and young mice (Figure 1b). Furthermore, the number of γδT cells was decreased in the spleen of aged mice; therefore, the observed increase in the number of γδT cells was specific to the lungs (Figure 1c).

**Figure 2:**
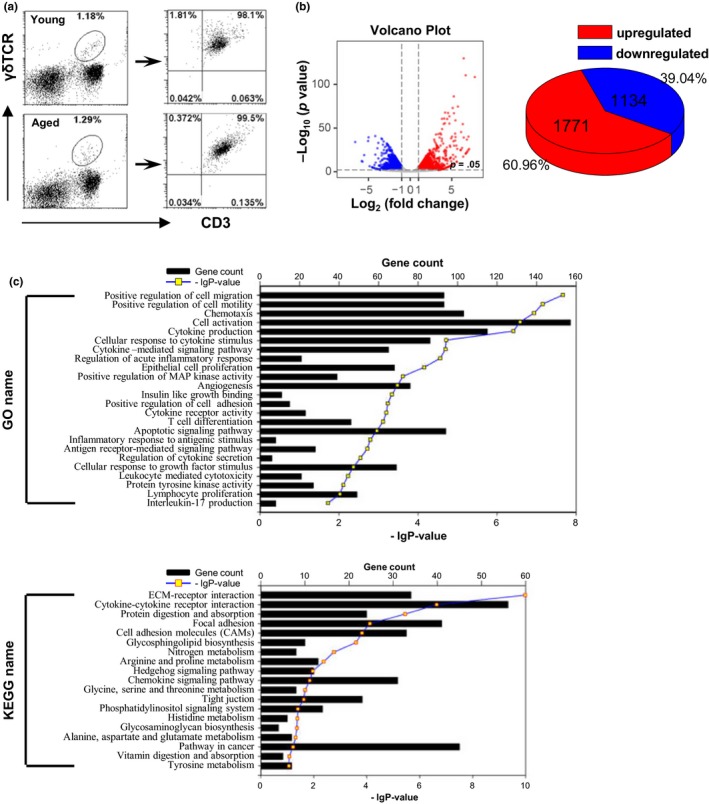
The gene expression of lung γδT cells was distinguished between aged and young mice. (a) γδT cells (CD3^+^ γδTCR^+^) were purified from lung MNCs (20 mice/sample) and detected using FACS. Purified γδT cells were analyzed through mRNA sequencing. (b) The Volcano plots based on the fold change and *p* value showed the differential expression of the indicated genes. The two vertical lines correspond to a twofold change in expression. The horizontal line indicates *p* = .05. Red plots represent the upregulated genes. Blue plots represent the downregulated genes. The pie chart shows the distribution of DEGs in the aged group compared with young group. (c) The list of DEGs was converted into Entrez‐IDs for GO and KEGG analyses with r 3.2.3 using the library gostats 2.34.0 and the R bioconductor genomewide mouse annotations from the package org.Mm.eg.db (version 3.3.0). DEGs, differentially expressed genes; FACS, fluorescence‐activated cell sorting; GO, Gene Ontology; KEGG, Kyoto Encyclopedia of Genes and Genomes; MNCs, mononuclear cells

Subsequently, lung‐resident γδT cells were purified (Figure 2a) and analyzed through mRNA sequencing. There were 2,905 differentially expressed genes (DEGs) between the aged and young mice, with 1,771 upregulated genes (60.96%) and 1,134 downregulated genes (39.04%; Figure 2b). Gene Ontology enrichment analysis showed that these DEGs altered in lung γδT cells of aged mice were mainly enriched in 24 biological pathways including cell activation, cytokine production, chemotaxis, positive regulation of cell migration and motility, cellular responses to cytokine, apoptotic signaling pathway, leukocyte mediated cytotoxicity, regulation of cytokine secretion, and IL‐17 production (Figure 2c). According to the Kyoto Encyclopedia of Genes and Genomes (KEGG) enrichment analysis, the DEGs were significantly enriched in cytokine–cytokine receptor interaction, pathway in cancer, focal adhesion, extracellular matrix (ECM)‐receptor interaction, cell adhesion molecules, biosynthesis, and metabolism (Figure 2c). These results indicated that aging caused significant alterations in lung‐resident γδT cells with changed immune functions.

### 2.2 Aged lung‐resident γδT cells predominantly produced IL‐17A

**Figure 3:**
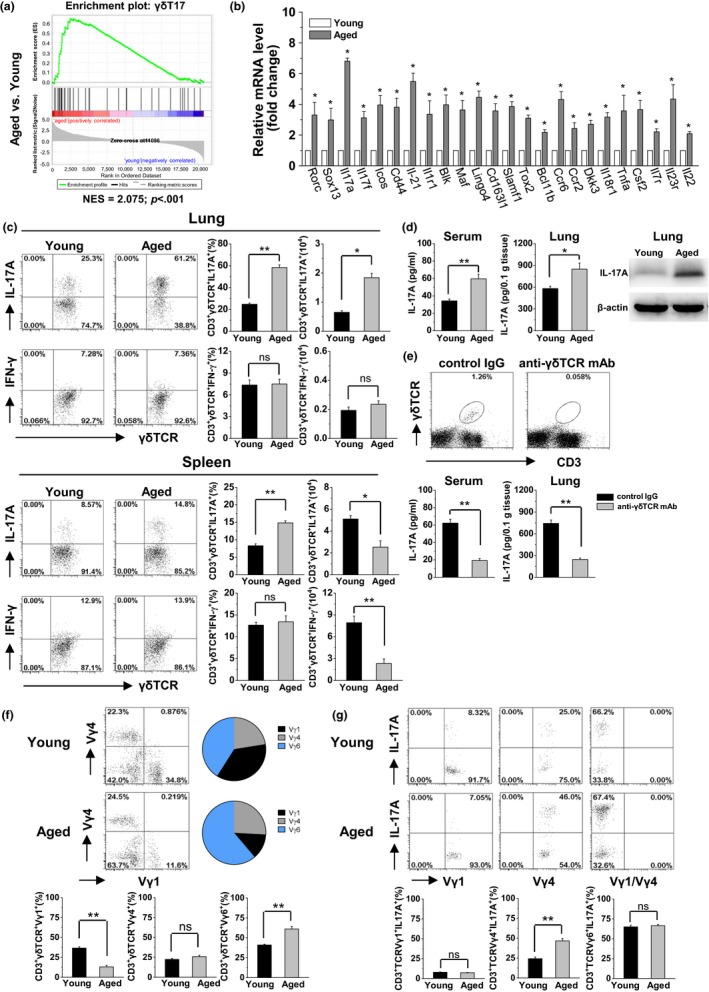
γδT17 cells were predominant in the lungs with high levels of IL‐17A production in aged mice. (a) According to the DEGs in lung γδT cells, GSEA from the aged versus young mice with normalized enrichment scores for γδT17 cell gene signatures. NES and *p* value are shown. (b) The mRNA expression levels of selected DEGs in the purified γδT cells (CD3^+^ γδTCR^+^) were measured using real‐time PCR (*n* = 3). (c) The MNCs were isolated from lung and spleen and analyzed using FACS. The CD3^+^ γδTCR^+^ cells were gated to analyze the frequency and number of CD3^+^ γδTCR^+^ IL‐17A^+^ cells (γδT17) and CD3^+^ γδTCR^+^ IFN‐γ^+^ cells (γδT1). There were six mice in each group. (d) The expression levels of IL‐17A protein in the serum were detected using ELISA (*n* = 3). In lung tissue, these levels were detected through ELISA and Western blotting. The results are representative of three independent experiments. (e) Inhibition of γδT cells was performed through injection of anti‐γδTCR mAb (UC7‐13D5, 200 μg/mouse, i.p.) twice every 3 days. Three days after the treatment, CD3^+^ γδTCR^+^ cells stained positively with a different monoclonal antibody against γδTCR (GL3) were analyzed using FACS. The expression levels of IL‐17A protein in the serum and lung tissue were detected using ELISA (*n* = 3). (f) Usage of the Vγ chain (Vγ1, Vγ4, and Vγ6) was analyzed for the lung γδT cells (CD3^+^ γδTCR^+^). Vγ6^+^ γδT cells were considered as Vγ1^‐^Vγ4^‐^ in the lungs. (g) Each γδT‐cell subset (CD3^+^ γδTCR^+^ Vγ1^+^, CD3^+^ γδTCR^+^ Vγ4^+^, and CD3^+^ γδTCR^+^ Vγ1^‐^Vγ4^‐^) was gated to analyze the production of IL‐17A. There were six mice in each group. The data are shown as the mean ± *SEM*. Student's *t* test was used. *p < .05, **p < .01. DEGs, differentially expressed genes; ELISA, enzyme‐linked immunosorbent assay; FACS, fluorescence‐activated cell sorting; GSEA, gene set enrichment analysis; mAb, monoclonal antibody; NES, normalized enrichment scores

The difference in cytokine production and regulation of cytokine secretion (Figure 2c) indicated that γδT‐cell subsets may be distinguished in aged mice (Figure 2c). Enrichment of 43 signature genes expressed by γδT17 cells showed that lung γδT cells of aged mice positively correlated with these genes when compared with the young mice (Figure 3a). Higher levels of mRNA expression of 24 indicated genes including Rorc, Il17a, Cd44, Ccr6, and Il23r further confirmed the predominant γδT17 cells in aged mice (Figure 3b). A higher percentage (>60%) and number of IL‐17A^+^γδT cells (γδT17) were present in the lungs of aged mice, while there was no difference in the percentage and number of IFN‐γ^+^ γδT cells (γδT1) in the lungs between aged and young mice (Figure 3c). In the spleen of aged mice, the number of both γδT17 and γδT1 cells decreased (Figure 3c). Distinguished from lung γδT cells, low levels of IL‐17A (positive cells <5%), and high levels of IFN‐γ (positive cells >20%) were observed for lung CD4^+^T and NKT cells in aged mice, which was similar to the spleen CD4^+^T and NKT cells (Figure S1).

The expression levels of IL‐17A protein in the serum and lungs were markedly higher in aged mice than in young mice (Figure 3d). To assess the importance of γδT in producing IL‐17A in aged mice, administration of anti‐γδTCR mAb was performed, which effectively inhibited γδT cells with the loss of γδT cells in the lungs, and subsequently reduced IL‐17A levels in the serum and lungs (Figure 3e). These results indicated that lung‐resident γδT cells predominantly produced IL‐17A in aged mice. Further, the lung γδT cells were detected with their Vγ expression to identify the producer of IL‐17. The frequency of Vγ1^+^ γδT cells was significantly lower in the aged mice (12.97 ± 1.85%) than in the young mice (36.62 ± 1.61%), while the frequency of Vγ1^−^Vγ4^−^ (considered as Vγ6^+^ in the lungs) γδT cells was significantly higher in aged mice (61.15 ± 3.29%) than in young mice (40.95 ± 1.06%). There was no difference observed in the frequency of Vγ4^+^ γδT cells between the aged mice (25.88 ± 1.63%) and young mice (22.43 ± 1.27%; Figure 3f). Among these γδT‐cell subsets, Vγ1^+^ γδT cells showed weak ability, whereas Vγ6^+^ γδT cells showed strong ability for the production of IL‐17A in the aged mice similar to the young mice (Figure 3g). Noticeably, the production of IL‐17A by Vγ4^+^ γδT cells was significantly increased in the aged mice (Figure 3g). These results indicated Vγ4^+^ and Vγ6^+^ γδT cells were the main producers of IL‐17A in the lungs of aged mice.

### 2.3 Aged mice were more resistant to the development of lung melanoma via enhancement of lung‐resident γδT17 cells

**Figure 4:**
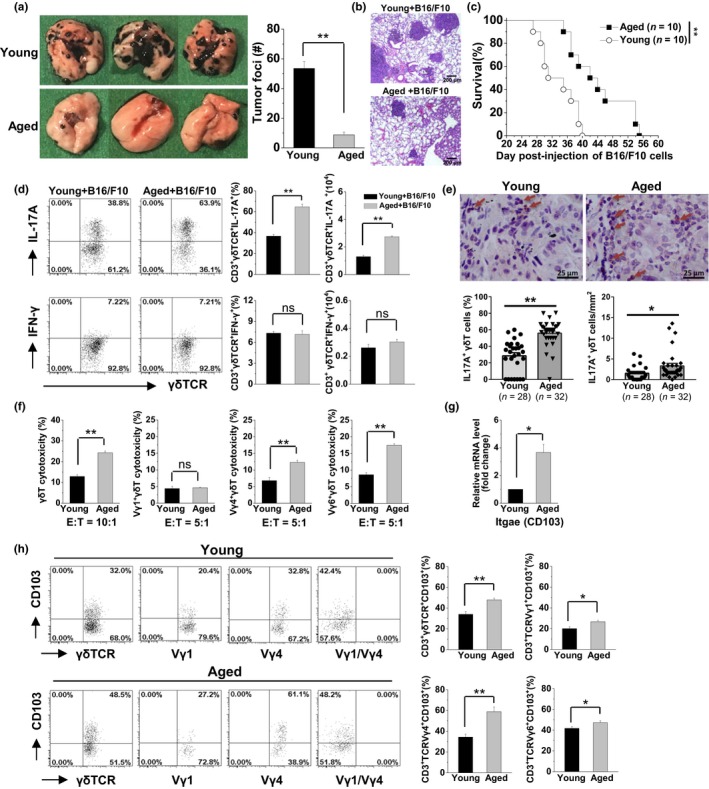
Higher anti‐tumor activity of γδT17 cells was associated with resistance to B16 melanoma metastasis in the lungs of aged mice. Young and aged mice were challenged with B16/F10 cells (1 × 10^5^ cells/mouse, i.v.). On day 21 after the B16/F10 challenge, the lungs were analyzed. (a) The graphs showed the tumor nodules in the lungs and the total number of tumor foci was calculated. Data are shown as mean ± *SEM*. Student's *t* test was used. **p < .01. (b) The lung samples were collected for hematoxylin and eosin staining. (c) The survival rate of B16/F10 challenged mice was calculated and analyzed using the Kaplan–Meier method. (d) On day 21 after the B16/F10 challenge, the CD3^+^ γδTCR^+^ cells were gated to analyze the frequency and number of CD3^+^ γδTCR^+^ IL‐17A^+^ cells (γδT17) and CD3^+^ γδTCR^+^ IFN‐γ^+^ cells (γδT1) in the lungs. There were six mice in each group. (e) Infiltrated γδT 17 cells in the lung cancer tissues in the aged patients compared with younger patients through histochemical analysis. γδTCR^+^ cells were shown as brown, IL‐17A^+^ cells were shown as red, γδTCR^+^ IL‐17A^+^ double‐positive cells were shown as brown‐red. The double‐positive cells of γδTCR^+^IL‐17A^+^ were indicated by the arrows. The percentages and numbers of γδTCR^+^IL‐17A^+^cells were calculated in each sample. (f) The cytotoxicity of purified lung γδT cells (CD3^+^ γδTCR^+^) against B16/F10 cells (E:T = 10:1), purified lung Vγ1^+^γδT cells (CD3^+^ γδTCR^+^ Vγ1^+^), Vγ4^+^γδT cells (CD3^+^ γδTCR^+^ Vγ4^+^), and Vγ6^+^γδT cells (CD3^+^ γδTCR^+^ Vγ1^‐^ Vγ4^‐^) against B16/F10 cells (E:T = 5:1). Data are shown as the mean ± *SEM* from triplicates of one of the three independent experiments. (g) The mRNA expression levels of CD103 in the purified γδT cells (CD3^+^ γδTCR^+^) were measured using real‐time PCR. (h) The expression levels of CD103 on each lung γδT‐cell subset were detected through flow cytometry analysis. The data are shown as the mean ± *SEM*. Student's *t* test was used. **p < .01. *p < .05. PCR, polymerase chain reaction; TCR, T‐cell antigen receptor

As previously reported (Martin‐Orozco et al., 2009), poorly immunogenic B16/F10 melanoma that colonizes to the lung by intravenous injection can be used to study the immune surveillance in the lung. Here, the immune function of lung‐resident γδT cells was investigated in aged mice using the B16 lung melanoma metastasis model. The same number of tumor cells colonized the lung tissue 48 hr after challenge with B16/F10 cells, indicating that aging did not affect the ability of B16/F10 to colonize the lungs (Figure S4). However, on day 21 after the challenge with B16/F10 tumor cells, the aged mice exhibited smaller tumors in size and markedly decreased numbers of tumor foci in the lungs compared with the young mice (Figure 4a,b). Moreover, the mean survival time of the aged mice (44.30 ± 2.43 days) was significantly longer than that of the young mice (33.70 ± 1.53 days; Figure 4c), indicating that the aged mice were resistant to the development of B16/F10 melanoma in the lungs.

After the B16/F10 melanoma challenge, the percentage and number of γδT17 cells in the lungs of aged mice were significantly higher than that in young mice, but this was not observed for the γδT1 cells (Figure 4d). Also, low levels of IL‐17A and high levels of IFN‐γ production in lung CD4^+^T and NKT cells were detected in the aged mice after the B16/F10 melanoma challenge (Figure S2). In addition, more infiltrated γδT17 cells were detected in the lung cancer tumor tissues of aged patients compared with younger patients, as shown by the higher percentages of IL‐17^+^γδT cells and the increased absolute numbers of IL‐17^+^γδT cells in the aged patients (Figure 4e). Furthermore, the anti‐tumor role of these increased γδT17 cells in aged mice was investigated. An in vitro cytotoxicity assay showed that purified lung γδT cells from aged mice were markedly more cytotoxic to B16/F10 tumor cells than that from young mice. Notably, the cytotoxicity of Vγ4^+^γδT cells and Vγ6^+^γδT cells was significantly higher in the aged mice especially Vγ6^+^γδT cells, but not Vγ1^+^γδT cells (Figure 4f), indicating the higher cytotoxicity of IL‐17‐producing γδT‐cell subsets in the aged mice compared with young mice. Higher expression of CD103—a molecule necessary for the immune synapse between γδT and tumor cells (Peters et al., 2019)—further indicated the enhanced anti‐tumor activity of lung γδT cells in aged mice (Figure 4g). The markedly higher expression levels of CD103 were observed on the lung‐resident Vγ4^+^γδT‐cell and Vγ6^+^γδT‐cell subsets of the aged mice (Figure 4h), further confirming their enhanced cytotoxicity against tumor cells.

**Figure 5:**
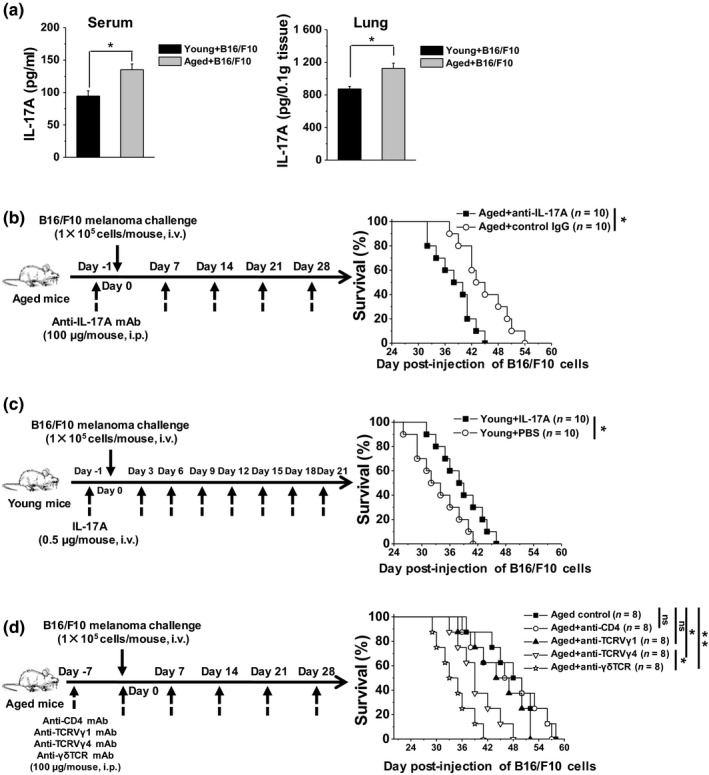
Aged mice exhibited higher anti‐tumor activity dependent on IL‐17A in the lungs. (a) On day 21 after the B16/F10 challenge (1 × 10^5^ cells/mouse, i.v.), the expression levels of IL‐17A protein in the serum and lung tissue were detected using ELISA (*n* = 3). The data are shown as the mean ± *SEM*. Student's *t* test was used. *p < .05. (b) Anti‐IL‐17A mAb was injected into the aged mice 1 day prior to injection of B16/F10 cells (1 × 10^5^ cells/mouse, i.v.). Additional injections were performed every 7 days. (c) IL‐17A was injected into young mice 1 day prior to injection of B16/F10 (1 × 10^5^ cells/mouse, i.v.). Additional injections were performed every 3 days. (d) Anti‐CD4 mAb, anti‐TCRVγ1, anti‐TCRVγ4, and anti‐γδTCR antibodies were injected i.p. into the aged mice 7 days prior to injection of B16/F10 cells (1 × 10^5^ cells/mouse, i.v.). Additional injections were performed every 7 days. The survival rate of B16/F10‐challenged mice was calculated and analyzed using the Kaplan–Meier method. *p < .05, **p < .01. ELISA, enzyme‐linked immunosorbent assay; mAb, monoclonal antibody: TCR, T‐cell antigen receptor

Markedly higher levels of IL‐17A in the serum and lung were observed in aged mice after the B16/F10 melanoma challenge (Figure 5a). Neutralization of IL‐17A using an anti‐IL‐17A mAb in aged mice significantly reduced the mean survival time from 45.10 to 38.20 days, demonstrating the critical anti‐tumor role of IL‐17A in the development of B16/F10 melanoma in aged lungs (Figure 5b). Furthermore, IL‐17A supplementation significantly enhanced anti‐tumor responses in young mice, prolonging the mean survival from 33.60 to 38.50 days (Figure 5c). Inhibition of γδT cells using an anti‐γδTCR mAb in aged mice significantly reduced the mean survival time from 48.63 to 34.38 days after the B16/F10 melanoma challenge, but not the depletion of CD4^+^T cells by anti‐CD4 mAb treatment (Figure 5d). Depletion of Vγ4^+^γδT cells but not Vγ1^+^γδT cells reduced the mean survival time in the challenged aged mice to 39.75 days, indicating the important role of the IL‐17A producer. The markedly shortened survival time in the anti‐γδTCR mAb‐treated aged mice compared with the anti‐TCRVγ4‐treated aged mice demonstrated that the Vγ6^+^γδT cells with high ability to produce IL‐17A also exhibited important anti‐tumor activity in the development of B16/F10 melanoma in aged lungs (Figure 5d). These results indicated that the alteration in lung‐resident γδT cells with enhanced production of IL‐17A accounted for the resistance to the development of B16/F10 melanoma in the lungs of aged mice.

### 2.4 Aged lung‐resident γδT17 cells exhibit intrinsic anti‐tumor activity independently of commensal microbiota

**Figure 6:**
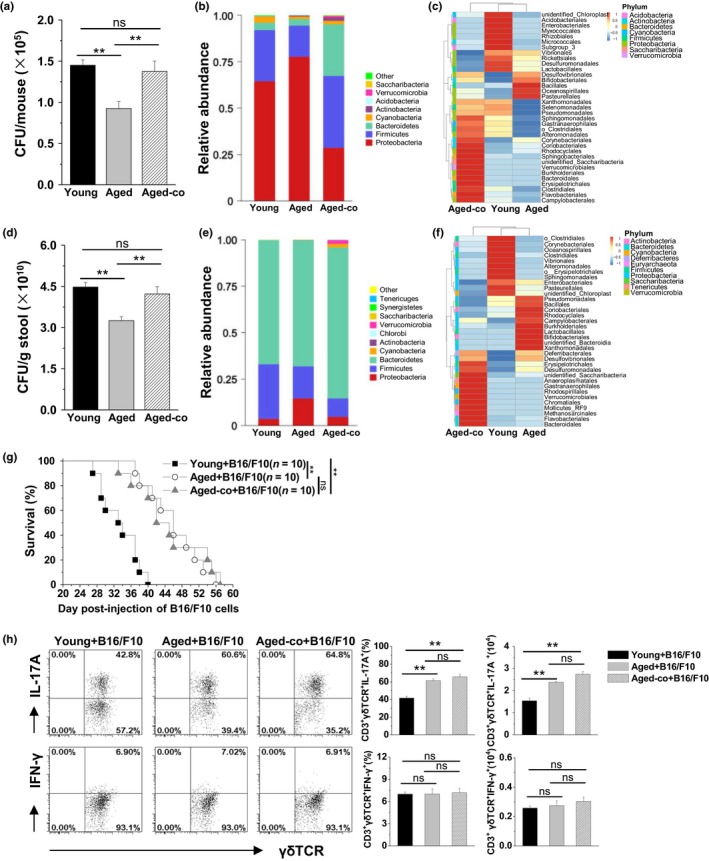
γδT cells showed intrinsic anti‐tumor activity with higher levels of IL‐17 production in the co‐housed aged mice independent of the alterations in the load and composition of commensal microbiota. In the co‐culture group, the aged mice were co‐housed with the young mice for 4 weeks. Bacterial loads were measured using BAP culture in the upper respiratory tract (a) and stool (d) from the co‐cultured aged mice compared with the control mice (*n* = 3/group). The data are shown as the mean ± *SEM*. Analysis of variance (one‐way ANOVA) was used. **p < .01. Relative abundance (b) and a clustering map (c) for the bacteria in the upper respiratory tract were determined through 16S rRNA analysis (3 samples/group, 10 mice/sample). Relative abundance (e) and a clustering map (f) for the bacteria in the stool were determined through 16S rRNA analysis (3 mice/group). (g) The co‐housed mice were challenged with B16/F10 cells (1 × 10^5^ cells/mouse, i.v.). The survival rates were calculated and analyzed using the Kaplan–Meier method. **p < .01. (h) On day 21 after the B16/F10 challenge, the MNCs isolated from the lungs were analyzed using FACS. The CD3^+^ γδTCR^+^ cells were gated to analyze the frequency and number of IL‐17A^+^ CD3^+^ γδTCR^+^ cells (γδT17) and IFN‐γ^+^ CD3^+^ γδTCR^+^ cells (γδT1). The data are shown as the mean ± *SEM* (*n* = 6). Analysis of variance (one‐way ANOVA) was performed. **p < .01. BAP, blood agar plate; FACS, fluorescence‐activated cell sorting; MNCs, mononuclear cells; TCR, T‐cell antigen receptor

We previously demonstrated that commensal microbiota modulated tumoral immune surveillance in lungs through a γδT17 immune cell‐dependent mechanism in young mice (Cheng et al., 2014). As shown in Figure 6a,d, the bacterial loads in the upper respiratory tract and stool were markedly lower in aged mice versus young mice. Thus, we used the 16S rRNA assay to analyze the bacterial composition. In the upper respiratory tract of aged mice, we observed a low frequency of *Firmicutes* and *Cyanobacteria*, and a high frequency of *Proteobacteria* and *Actinobacteria* in the overall composition (Figure 6b,c). In the stool of aged mice, we observed a low frequency of *Firmicutes*, and a high frequency of *Proteobacteria*, *Bacteroidetes*, and *Actinobacteria* (Figure 6e,f). Interestingly, following co‐housing of aged mice with young mice for 4 weeks, the bacterial load in the upper respiratory tract and stool was significantly increased to reach that of young mice (Figure 6a,d). The bacterial composition in the upper respiration tract and stool was also markedly altered in the co‐cultured aged mice, distinguished from aged mice and young mice (Figure 6b,c,e). However, in these co‐cultured aged mice with normal body weight and lung index, the number of lung γδT cells was not altered (Figure S3). Consequently, the co‐cultured aged mice were resistant to the development of B16/F10 melanoma in the lungs, with the mean survival time similar to aged mice, but much longer than that in young mice (Figure 6g). In the co‐cultured aged mice, the lung γδT cells were still characterized by enhanced production of IL‐17A but not IFN‐γ (Figure 6h). These results indicated that the anti‐tumor activity of γδT17 cells was intrinsic in aged mice independently of alterations in the load and composition of commensal microbiota.

## 3 DISCUSSION

Despite the important role of γδT cells in innate and adaptive immunity, information regarding the effect of aging on the function of γδT cells in vivo is limited. In this study, we performed a comprehensive evaluation of lung‐resident γδT cells in aged mice compared with young mice. Aging intrinsically induced γδT cells to predominantly produce IL‐17A, which played a critical role in resistance to the development of lung melanoma in the aged mice. These findings may explain the lower incidence and slower progression of lung cancer observed in the elderly population.

Physiological aging is accompanied by a decline in the normal functions of the immune system including hematopoietic stem cells, and innate and adaptive immune cells. In this study, we demonstrated that γδT cells were significantly altered in aged lungs, with increased numbers and distinct gene expression (Figures 1b,2, and 3). Changes in multiple levels (e.g., cellular, tissue, and systemic) may contribute to alterations in the immune cells of aged individuals (Oishi & Manabe, 2016). Firstly, changes in cellular response to cytokine and growth factor stimuli and in cellular metabolism were determined in aged lung‐resident γδT cells (Figure 2c). Secondly, accumulation of cell debris and increased levels of pro‐inflammatory cytokines TNF‐α and IL‐6, surfactant proteins and lipids (e.g., SP‐A and SP‐D) and several complement components has been observed in aged lungs with a relatively oxidized tissue microenvironment in both mice and humans. This may modify mucosal immune responses in the elderly population (Moliva et al., 2014). Thirdly, systemic changes in metabolic and hormonal signals likely contribute to the development of chronic inflammation in the aged (Oishi & Manabe, 2016). Aged lung γδT cells were intrinsically altered independently of the load and composition of commensal microbiota (Figure 6), indicating the unimportance of this microenvironment factor. However, in young mice, the microbiota modulated the γδT‐cell immune response (Cheng et al., 2014). It was also found that segmented filamentous bacteria only induced the non‐Tfh cells to upregulate Bcl‐6 in the young but not the middle‐aged group, since the accumulated Tfh cells in middle‐aged mice had an effector phenotype induced at an earlier age (Teng et al., 2017). It was speculated that the γδT cells intrinsically acquired an irreversible effector function with age similar to Tfh cells. Regarding the other immune cells, IFN‐γ‐producing NK cells in LPS‐challenged lungs were decreased in aged mice as compared with young mice in an IL‐12‐dependent manner. However, the innate IL‐12/IFN‐γ axis is not intrinsically defective in lungs of aged mice, but rather suppressed by the enhanced production of mononuclear phagocyte‐derived IL‐10 (Chiu et al., 2008). Differently, in this study IFN‐γ‐producing NK cells were not changed in aged mice as compared with young mice in the normal and in B16/F10 melanoma‐challenged lungs (data not shown). Notably, the old naïve CD8^+^T cells could acquire normal response in the adult environment (Jergovic, Smithey, & Nikolich‐Zugich, 2018). In our future research, the intrinsic alteration of γδT17 cells in aged mice will be further confirmed by transferring aged γδT cells into young mice to absolutely exclude changes in the tissue microenvironment.

Consistently with previously reported findings (Cheng et al., 2014; Kisielow & Kopf, 2013), the present study demonstrated the beneficial role of IL‐17A derived from γδT cells in tumor surveillance for lung cancer (Figure 5). IL‐17 exerted anti‐tumor functions by recruiting neutrophils, NK cells, and CD4^+ ^and CD8^+ ^T cells to tumor tissue and enhancing NK cell and cytotoxic T lymphocyte (CTL) activation (Qian et al., 2017). IL‐17A derived from γδT17 cells plays an important role in chemotherapy‐induced anticancer immune responses by preceding the accumulation of Tc1 CTLs within the tumor bed (Ma et al., 2011). Here, aged lung γδT cells with predominant production of IL‐17A directly exerted their higher cytotoxicity to tumor cells (Figure 4e). The important inflammatory cytokine IL‐17 acts as double‐edged sword in anti‐tumor immunity and tumorigenesis, since IL‐17 also facilitated tumor angiogenesis and enhanced tumor immune evasion (Murugaiyan & Saha, 2009; Qian et al., 2017). As recently reported, activated lung‐resident γδT cells that produced IL‐17 and other effector molecules promoted neutrophil infiltration and lung adenocarcinoma development induced by Kras mutation and p53 loss (Jin et al., 2019). It was speculated that the inconsistent observation of γδT17 cell function may be related to the lung cancer model, since the lung adenocarcinoma was different from the metastatic lung melanoma. Additionally, age is also an important element. The promotion of lung adenocarcinoma development by γδT17 cells was observed in the adult young mice (8–15 weeks old), in which γδT17 cells were driven to proliferate and activate by the increased bacterial burden with the lung cancer development (Jin et al., 2019); while the suppression of metastatic lung melanoma by γδT17 cells was in the aged mice (20–24 months old), in which aged lung γδT cells were intrinsically altered independently of the load and composition of commensal microbiota (Figure 6). Moreover, other immune cells such as neutrophils that owned declined functions with age may affect the responses of γδT17 cells during the development of lung cancer (Boe, Boule, & Kovacs, 2017).

Targeting of IL‐17A derived from Th17 cells is beneficial in experimental lung cancer by inhibiting lung tumor‐infiltrating T regulatory cells and inducing IFN‐γ‐producing CD4^+^ T cells in the presence of T‐bet (Reppert et al., 2011). In aged mice, a low expression level of IL‐17A was observed in lung CD4^+^T cells (Figure S1 and S2), implying that they may not be important in this model. However, higher expression levels of IFN‐γ were detected in aged CD4^+^T and NKT cells (Figure S1 and S2), which roles in the aged deserved further investigation.

In addition to lung cancer, γδT cells are involved in several lung diseases including bacterial, viral and fungal infections, allergic disease, inflammation, and fibrosis (Cheng & Hu, 2017). IL‐17A produced by lung‐resident γδT cells suppresses allergic inflammation by inhibiting Th2‐driven inflammation and eosinophil influx (Murdoch & Lloyd, 2010; de Oliveira Henriques & Penido, 2012). During bacterial infection, IL‐17A derived from activated lung γδT cells recruits neutrophils, induces mature granuloma formation, or induces Th17 immune responses to perform their defense functions (Bai et al., 2017; Okamoto Yoshida et al., 2010; Ye et al., 2001). During viral infections, IL‐17A derived from activated lung γδT cells inhibits virus replication (Tu et al., 2011). On the other hand, enhancement of allergic airway inflammation and anti‐viral inflammation has also been related to lung‐resident γδT cells (Cheng & Hu, 2017). The influence of these intrinsic alterations of lung‐resident γδT cells on the progression of these lung diseases in the aged population requires further investigation, especially the development of chronic obstructive pulmonary disease and increased susceptibility to numerous pulmonary infections (e.g., influenza, pneumococcal pneumonia, tuberculosis) in the elderly.

Noticeably, old age is associated with reduced function of the immune system, including both the innate and adaptive immune cells. In addition to γδT cells, the number of NK cells was decreased in the lungs of aged mice (Figure 1b), and the number of both γδT17 and γδT1 cells was decreased in the spleen of aged mice (Figure 3c). We also observed the decreased percentage and number of alveolar macrophages in the lungs of aged mice (data not shown). The immune responses to other antigens or pathogens warrant further investigation to explore the dysfunction of the immune system in the aged mice.

In summary, we demonstrated that lung‐resident γδT cells were intrinsically altered, predominantly producing IL‐17A in aged mice, which played a critical role in controlling lung melanoma in the aged. These findings may enhance our understanding of the lower incidence and slower progression of lung cancer in elderly populations, and the potential use of γδT cells in anti‐tumor immunotherapy. Considering the dramatic increase in the aging population, the control of age‐related immune inflammation is of great importance and warrants further investigation.

## 4 EXPERIMENTAL PROCEDURES

### 4.1 Mice

Female C57BL/6 mice were obtained from the Shanghai Experimental Center of the Chinese Science Academy (Shanghai, China). Young mice (10–16 weeks) and aged mice (20–24 months) were used. The aged mice (22 months) are equivalent to about 75 years old of human according to the lifespan of laboratory mice and the life expectancy of humans (Dutta & Sengupta, 2016). All mice were maintained under specific‐pathogen‐free and controlled conditions (22°C, 55% humidity, and a 12‐hr day/night rhythm), in accordance with the Guide for the Care and Use of Laboratory Animals granted by University of Science and Technology of China.

### 4.2 Induction and assessment of B16/F10 lung melanoma

B16/F10 cells (a mouse melanoma cell line) were obtained from the American Type Culture Collection (ATCC, Manassas, VA, USA) and maintained in DMEM (Gibco BRL) containing 10% heat‐inactivated fetal bovine serum (ExCell Biology). Mice were injected intravenously (i.v.) with 1 × 10^5^ B16/F10 cells. On day 21 after B16/F10 cell challenge, the number of metastatic lung foci was counted. An equal volume of phosphate‐buffered saline (PBS) alone (250 μl) was used as a control.

### 4.3 Isolation of mononuclear cells (MNCs)

As previously described (Wang et al., 2012), MNCs were isolated from the lungs and spleen via density gradient centrifugation using 40% and 70% Percoll solution (Gibco BRL).

### 4.4 Flow cytometry analysis

The surface phenotype assays and the intracellular cytokine assay were performed as described in supplemental materials and methods S1 (Cheng et al., 2014). The monoclonal antibodies (mAb) used for FACS are shown in Supplemental Table S1.

### 4.5 Gene set enrichment analysis (GSEA)

GSEA was performed using the gsea v2.2.4 software (Broad Institute, Cambridge, MA, USA) with default parameters, inclusion gene set size between 15 and 500, and phenotype permutation at 1,000 times. The γδT17 cell gene set was created for the GSEA analysis according to previously reported highly related genes, as shown in the supplemental materials and methods S1.

### 4.6 Histological examination

Lung samples were fixed in 10% neutral‐buffered formalin and embedded in paraffin. HE staining and histochemical analysis were performed as described in the Supplemental Materials and Methods S1.

### 4.7 In vitro cytotoxicity assay

The cytotoxicity of γδT cells against B16/F10 cells was measured using the CellTrace^™^ Far Red Kit (Invitrogen) according to the instructions provided by the manufacturer.

### 4.8 Detection of IL‐17A protein

The expression levels of IL‐17A protein in the serum and lung homogenate were detected using a mouse IL‐17A enzyme‐linked immunosorbent assay (ELISA) kit (eBioscience). Lung samples were homogenized in buffer containing Triton X‐100 and a protease inhibitor cocktail (Complete Mini; Roche). Anti‐IL17A antibody (clone TC11‐18H10, Abcam) was used to detect IL‐17A in the lungs through Western blotting, as presented in the Supplemental Materials and Methods S1.

### 4.9 IL‐17A treatment

Recombinant murine IL‐17A (PeproTech) was injected i.v. into young mice (0.5 μg/mouse) 1 day prior to the injection of B16/F10 cells. Additional injections were performed every 3 days. An equal volume of 250 μl PBS alone was used as a control.

### 4.10 Antibody neutralization

A recombinant anti‐mIL‐17A antibody (clone 17F3, BioXcell) was injected intraperitoneally (i.p.) into aged mice (100 μg/mouse) 1 day prior to the injection of B16/F10 cells. Additional injections were performed every week. Control mice received equal amounts of control immunoglobulin (Ig) G (clone MOPC‐21, BioXcell).

### 4.11 In vivo function experiments of γδT cells and CD4^+^T cells

To elucidate the functional role of γδT cells, anti‐γδTCR antibody (clone UC7‐13D5, BioXcell) was injected i.p., into aged mice (200 μg/mouse) every 3 days for two times, and then, serum and tissue IL‐17A were detected. Control mice received equal amounts of isotope control antibody (Armenian hamster IgG, BioXcell). For the survival analysis, anti‐CD4 mAb (clone YTS191, BioXcell), anti‐TCRVγ1 (clone 2.11, BioXcell), anti‐TCRVγ4 (clone UC3‐10A6, BioXcell), and anti‐γδTCR (clone UC7‐13D5, BioXcell) antibodies were injected i.p., into the aged mice (200 μg/mouse) 7 days prior to injection of B16/F10 (1 × 10^5^ cells/mouse, i.v.). Additional injections (200 μg/mouse) were performed every 7 days.

### 4.12 Statistical analysis

All data are shown as the mean ± standard error of the mean (*SEM*). Differences between individual data were analyzed using Student's *t* test or one‐way analysis of variance, as appropriate. Least significant difference tests (LSD, 0 < α < 1) were used for the post hoc tests. The mouse survival rate was analyzed using the Kaplan–Meier method. A *p* value < .05 was considered statistically significant.

## CONFLICT OF INTEREST

The authors have declared no conflict of interests.

## AUTHOR CONTRIBUTIONS

Min Cheng designed and performed all the experiments, analyzed data, and prepared the manuscript. Yongyan Chen prepared and revised the manuscript. Dake Huang performed the histological examination. Wen Chen performed the FACS experiments. Weiping Xu directed the result analysis and article writing. Yin Chen performed the sequencing data analysis. Guodong Shen established the lung tumor model. Tingjuan Xu performed the tumor cell line culture in vitro. Gan Shen supervised the study. Zhigang Tian directed the result analysis and article writing. Shilian Hu offered the financial support and supervised the study.
