# Supplementary material for: Intrinsically altered lung‐resident γδT cells control lung melanoma by producing interleukin‐17A in the elderly
Source: Aging Cell. 2020 Jan 5;19(2):e13099. doi: 10.1111/acel.13099 (PMC6996947; doi:10.1111/acel.13099)
Supplement: Supplementary file 1 [file ACEL-19-e13099-s001.docx]

**Intrinsically altered lung-resident γδT cells control lung melanoma by producing interleukin-17A in the elderly**

Min Cheng^1,2,3^, Yongyan Chen^4^, Dake Huang^5^, Wen Chen^1,2,3^, Weiping Xu^1,2^, Yin Chen^1,2^, Guodong Shen^1,2^, Tingjuan Xu^1,2^, Gan Shen^1,2^, Zhigang Tian^3,4^, Shilian Hu^1,2^

^1^ Gerontology Institute of Anhui Province, the First Affiliated Hospital of University of Science and Technology of China (Anhui Provincial Hospital), Hefei, 230001, China;

^2^ Anhui Provincial Key Laboratory of Tumor Immunotherapy and Nutrition Therapy, Hefei, 230001, China;

^3^ Cancer Immunotherapy Center, the First Affiliated Hospital of University of Science and Technology of China (Anhui Provincial Hospital), Hefei, 230001, China;

^4^ Institute of Immunology and The CAS Key Laboratory of Innate Immunity and Chronic Disease, School of Life Science and Medical Center, University of Science and Technology of China, Hefei 230027, China

^5^ Comprehensive Laboratory, School of Basic Medical Sciences, Anhui Medical University, Hefei, 230032, China;

Address correspondence and reprint requests to Min Cheng or Shilian Hu. Gerontology Institute of Anhui Province, the First Affiliated Hospital of University of Science and Technology of China (Anhui Provincial Hospital), Hefei, 230001, China; E-mail: [chengmin@ustc.edu.cn](mailto:chengmin@ustc.edu.cn) or hushilian@126.com.

**Supplemental Materials and Methods S1**

***Quantitative real-time polymerase chain reaction (PCR)***

Total RNA was extracted from the purified γδT cells (CD3^+^ γδTCR^+^) using a miRNeasy Mini Kit (QIAGEN, Duesseldorf, Germany). mRNA was reverse transcribed into cDNA in a 150 μl reaction volume containing the following reagents: 10 μg of total mRNA, 5 μmol/L oligdT, 0.5 mmol/L dNTPs, 30 μl of 5×buffer, 10 mmol/L DTT, 300 units of RNase inhibitor, 1500 units of M-MLV and distilled water (ultrapure, DNase and RNase free). The RT reaction was performed at 37°C for 50 minutes, followed by heating at 70°C for 15 minutes. The standard 50 μl volume reaction contained 25 μl 2×PCR buffer, 2 μl cDNA template, 0.4 μmol/L forward and reverse primers. Quantitative real-time PCR was performed using Roche Light Cycler 480 II (Roche Diagnostics, Germany). PCR reactions were performed using a total of 45 cycles consisting of a 15 s melt at 95°C, followed by a 30 s annealing at 60°C, 30 s extension at 72°C. Each sample was analyzed in triplicate for each target gene. Gene expression levels were quantified using the ΔΔCt method. Information on gene-specific primers is shown in Supplemental Table 2.

***Purification of γδT cells***

Isolated lung MNCs were stained with PerCP-CY5.5-conjugated anti-CD3e (clone 145-2C11, BD Pharmingen, San Diego, CA, USA), FITC-conjugated anti-γδTCR (clone GL3, eBioscience, San Diego, CA, USA), APC-conjugated anti-TCRVγ1 (clone 2.11, BioLegend, San Diego, CA, USA) and PE-CY7-conjugated anti-TCRVγ4 (clone UC3-10A6, eBioscience, San Diego, CA, USA). Subsequently, γδT cells (CD3^+^ γδTCR^+^), Vγ1^+^ γδT cells (CD3^+^ γδTCR^+^ Vγ1^+^), Vγ4^+^ γδT cells (CD3^+^ γδTCR^+^ Vγ4^+^) and Vγ6^+^ γδT cells (CD3^+^ γδTCR^+^ Vγ1^-^ Vγ4^-^) were sorted using a FACS Aria II flow cytometer (Becton Dickinson, Franklin Lakes, NJ, USA). The purity of the separated cells was >95%.

***mRNA sequencing***

Total RNA was extracted from the purified γδT cells (CD3^+^ γδTCR^+^) using a miRNeasy Mini Kit (QIAGEN, GmBH, Germany). RNA quality was confirmed with a 2100 Bioanalyzer from Agilent. cDNA was synthed and amplificated with the SMARTer Ultra Low RNA Kit (Clontech, Mountain View, CA,USA) following the manufacturer’s instruction. A TrueSeq DNA library preparation kit (Illumina, USA) was used to prepare the RNA sequencing library according to the manufacturer's protocol. Illumina paired-end 150 bp sequencing was performed on a HiSeq2500 apparatus (Illumina, USA). Sequencing raw reads were preprocessed by filtering out rRNA reads, sequencing adapters, short-fragment reads and other low-quality reads using Seqtk. Hisat2 (version:2.0.4) was used to map the cleaned reads to the mouse GRCm38.p4 (mm10) reference genome with two mismatches. After genome mapping, Stringtie (version:1.3.0) was run with a reference annotation to generate FPKM values (Fragments Per Kilobase of exon model per Million mapped reads) for known gene models. Differentially expressed genes were identified using edgeR. The p-value significance threshold in multiple tests was set by the false discovery rate (FDR).

***Gene Ontology (GO) and KEGG pathway analysis***

GO and KEGG pathway analysis were performed as previously described ([Ashburner *et al.* 2000](#_ENREF_1); [Kanehisa & Goto 2000](#_ENREF_5)). Differentially expressed genes (DEGs) (fold change≥2) were converted to Entrez-IDs for GO and KEGG analysis with R 3.2.3 software using the library GO stats 2.34.0 (Author: Adrian Alexa, Jorg Rahnenfuhrer ) and the R Bioconductor genome-wide mouse annotations from package org.Mm.eg.db (version 3.4.0) (Author: Marc Carlson). The results were ranked according to the p values (p<0.05).

***Gene set enrichment analysis (GSEA)***

The mouse γδT17 cell gene set was not found in the Broad Institute database, so we identified some genes highly related to γδT17 cells by references, and established a gene set by ourselves for the GSEA analysis. Totally there are 43 genes including Hes1, Rela, Relb, Ltbr, Notch1, Notch4, Tgfbr2, Tgfbr3, Thy1, Klf3, Il6ra, Etv5, Tcf7, Il7r, Il17ra, Smad3, Il22, Sox4, Notch3, Rorc, Sox13, Notch2, Il17a, Il17f, Icos, Cd44, Il23r, Il1r1, Blk, Maf, Lingo4, Cd163l1, Slamf1, Tox2, Bcl11b, Ccr6, Ccr2, Dkk3, Il18r1, Tnfa, Csf2, Itgae, Il21. Gene sets that met the false discovery rate 0.25 criterion were considered. Normalized enrichment scores (NES) and p values are shown.

***Histological examination***

For histological examination, lung samples were fixed in 10% neutral-buffered formalin and embedded in paraffin. Sections of 4 μm thickness were stained with hematoxylin and eosin using the routine methods. Histochemical analysis was performed by anti-α-SMA (alpha smooth muscle actin) antibody (clone E184, Abcam, Cambridge, MA, USA), anti-CK8 (cytokeratin 8) antibody (clone EP1628Y, Abcam, Cambridge, MA, USA), anti-GFP (green fluorescent protein) antibody (clone EPR14104, Abcam, Cambridge, MA, USA) and DAB Peroxidase Substrate Kit (PV-6000, ZSGB-BIOTECH CO., LTD, Beijing, China). The human NSCLC tissue arrays including squamous cell carcinoma (Catalog number: HLug-Squ150Sur-02, OD-CT-RsLug01-009 TMA) and adenocarcinoma (Catalog number: HLug-Ade180Sur-01, HLug-Ade180Sur-03, HLug-Ade180Sur-06) were purchased from Shanghai Outdo Biotech Co., Ltd. (Shanghai, P. R. China). Anti-IL-17A polyclonal antibody (Catalog # PA5-79470, Invitrogen, Carlsbad, CA, USA), anti-human γδTCR antibody (clone 5A6.E9, , Invitrogen, Carlsbad, CA, USA) and double Staining Kit (Mo/HRP+Rb/AP, DS-0002, ZSGB-BIOTECH CO., LTD, Beijing, China) were used for the histochemical staining. Samples of aged patients with lung cancer (≥75 years old, n=32) and younger patients with lung cancer (≤50 years old, n=28) were selected and statistically analyzed for the infiltrated γδT17 cells. The information of these patients was shown in the supplemental Table 3. The sections were photographed using an Olympus IX73 microscope (Olympus, Tokyo, Japan).

***In vitro cytotoxicity assay***

The CellTrace^TM^ Far Red Kit (Invitrogen, Carlsbad, CA, USA) was used. Labeled B16/F10 cells (target cells, 1×10^4^/well) were incubated with γδT cells (effector cells, 1×10^5^/well), Labeled B16/F10 cells (target cells, 5×10^3^/well) were incubated with Vγ1^+^γδT cells (effector cells, 2.5×10^4^/well), Vγ4^+^γδT cells (effector cells, 2.5×10^4^/well) or Vγ6^+^γδT cells (effector cells, 2.5×10^4^/well) in a total volume of 200 μl RPMI 1640 medium with 10% heat-inactivated fetal bovine serum in round-bottomed 96-well plates at 37°C in 5% CO_2_ for 4 hours. PI (BD Pharmingen, San Diego, CA, USA) was added to the medium for another 15 min and CellTrace™ Far Red^+^ PI^+^ cells were analyzed by flow cytometry. Specific cytotoxicity was determined as follows: % specific lysis = (CT – TE) / CT ×100%.

***Western blotting***

Lung tissues were lysed in cell lysis buffer (Beyotime, China) with a pro-tease inhibitor cocktail (Complete Mini; Roche, Switzer-land) for 5 min in ice, and then centrifuged at 13,000 g for 5 min at 4°C. The concentration of total protein in the supernatant was measured by BCA Protein Assay Kit (Thermo Fisher Scientific, Waltham, MA, USA). After SDS-PAGE, proteins were transferred onto PVDF membranes (Millipore Corporation, Billerica, USA), and incubated with anti-IL17 antibody (clone TC11-18H10, Abcam, UK) over-night at 4°C. Membranes were washed with 0.1% (vol/vol) Tween 20 in TBS (pH 7.6) and incubated with a 1:2,500 dilution of horseradish peroxidase-conjugated anti-rat IgG (CST, Danvers, MA,USA) for 60 min at room temperature. Protein bands were visualized by ECL reaction (Pierce Biotechnology, Rockford, IL, USA).

***Blood agar plate culture***

As previously described ([Cheng *et al.* 2014](#_ENREF_3); [Cheng *et al.* 2017](#_ENREF_2)), 100 mg fresh stool was dissolved in 1 ml PBS, serially diluted, and coated on blood agar plates (9 cm, Hefei Tianda Diagnostic Reagent Co. Ltd., Hefei, China). To quantify the commensal bacteria burden in the upper respiratory tract, nasal washes were collected by back-flushing 2 ml sterile saline and plated with serial dilutions of 100 µl lavage fluid dissolved in 1 ml PBS ([Ichinohe *et al.* 2011](#_ENREF_4)). After 36 h of culture at 37°C, the colony-forming units of bacteria were measured.

***Analysis of bacterial diversity***

As previously described ([Cheng *et al.* 2014](#_ENREF_3); [Cheng *et al.* 2017](#_ENREF_2)), DNA was extracted from 0.18–0.22 g stool using a QIA amp DNA Stool Mini Kit (QIAGEN). DNA was extracted from nasal wash using a DNeasy Blood & Tissue Kit (QIAGEN) and lysozyme from chicken egg white (Sigma, St Louis, MO). The DNA was recovered with 30 µL of AE buffer. The 16S ribosomal RNA (rRNA) gene was analyzed to evaluate the bacterial diversity using Illumina Miseq (Novogene Bioinformatics Technology Co., Ltd, Beijing, China).

***Flow cytometry analysis***

For the surface phenotype assay, mononuclear cells (1 × 10^6^) were blocked with normal rat serum (10 μL/sample) for 30 min at 4°C, and then stained with the indicated for 30 min at 4°C in the dark. For the intracellular cytokine assay, freshly isolated cells were stimulated with 50 ng/mL phorbol 12-myristate 13-acetate (PMA, Sigma, St Louis, MO), 1 μg/mL ionomycin (Sigma, St Louis, MO) and treated with 10 μg/mL monensin (Sigma, St Louis, MO) for 4 h. The cells were labeled with surface markers, and then fixed and permeabilized using 100 μL of cytofix and cytoperm solution (eBioscience, San Diego, CA) according to the product manual. Then the cells were labeled with the indicated intracellular fluorescence-labeled mAbs for 30 min at 4°C in the dark. All data were acquired using a fluorescence-activated cell sorting (FACS)-Verse flow cytometer (Becton-Dickinson, Franklin Lakes, NJ, USA) and analyzed using the FlowJo software version 7.6.1 (Treestar, Ashland, OR, USA).

**References**

Ashburner M, Ball CA, Blake JA, Botstein D, Butler H, Cherry JM, Davis AP, Dolinski K, Dwight SS, Eppig JT, Harris MA, Hill DP, Issel-Tarver L, Kasarskis A, Lewis S, Matese JC, Richardson JE, Ringwald M, Rubin GM, Sherlock G (2000). Gene ontology: tool for the unification of biology. The Gene Ontology Consortium. *Nat Genet*. **25**, 25-29.

Cheng M, Chen Y, Wang L, Chen W, Yang L, Shen G, Xu T, Tian Z, Hu S (2017). Commensal microbiota maintains alveolar macrophages with a low level of CCL24 production to generate anti-metastatic tumor activity. *Scientific reports*. **7**, 7471.

Cheng M, Qian L, Shen G, Bian G, Xu T, Xu W, Hu S (2014). Microbiota modulate tumoral immune surveillance in lung through a gammadeltaT17 immune cell-dependent mechanism. *Cancer research*. **74**, 4030-4041.

Ichinohe T, Pang IK, Kumamoto Y, Peaper DR, Ho JH, Murray TS, Iwasaki A (2011). Microbiota regulates immune defense against respiratory tract influenza A virus infection. *Proceedings of the National Academy of Sciences of the United States of America*. **108**, 5354-5359.

Kanehisa M, Goto S (2000). KEGG: kyoto encyclopedia of genes and genomes. *Nucleic acids research*. **28**, 27-30.

Mathew R, Futterweit S, Valderrama E, Tarectecan AA, Bylander JE, Bond JS, Trachtman H (2005). Meprin-alpha in chronic diabetic nephropathy: interaction with the renin-angiotensin axis. *Am J Physiol Renal Physiol*. **289**, F911-921.

Miller PG, Bonn MB, McKarns SC (2015). Transmembrane TNF-TNFR2 Impairs Th17 Differentiation by Promoting Il2 Expression. *J Immunol*. **195**, 2633-2647.

Sartoretto S, Gemini-Piperni S, da Silva RA, Calasans MD, Rucci N, Pires Dos Santos TM, Lima IBC, Rossi AM, Alves G, Granjeiro JM, Teti A, Zambuzzi WF (2018). Apoptosis-associated speck-like protein containing a caspase-1 recruitment domain (ASC) contributes to osteoblast differentiation and osteogenesis. *Journal of cellular physiology*.

**Supplemental Table 1. The monoclonal antibodies for FACS**

| **Fluorescein** | **Antibody** | **Clone number** | **Manufacturer** | **Isotype control** |
| --- | --- | --- | --- | --- |
| FITC | anti-CD4 | RM4-5 | BD Pharmingen | Rat IgG2α, κ |
| FITC | anti-NK1.1 | PK136 | Biolegend | Ms IgG2a, κ |
| FITC | anti-CD19 | 1D3 | BD Pharmingen | Rat IgG2a, κ |
| FITC | anti-TCR-γδ | GL3 | eBioscience | ArH IgG |
| FITC | anti-CD103 | M290 | BD Pharmingen | Rat IgG2a, κ |
| PE | anti-CD8α | 53-6.7 | BD Pharmingen | Rat IgG2α, κ |
| PE | anti-TCR-γδ | GL3 | eBioscience | ArH IgG |
| PE | anti-IL-17A | TC11-18H10 | BD Pharmingen | Rat IgG1, κ |
| PE-CY7 | anti-NK1.1 | PK136 | Biolegend | Ms IgG2a, κ |
| PE-CY7 | anti-TCRVγ4 | UC3-10A6 | eBioscience | ArH IgG |
| PerCP-CY5.5 | anti-NK1.1 | PK136 | Biolegend | MS IgG2α, κ |
| PerCP-CY5.5 | anti-CD3e | 145-2C11 | BD Pharmingen | AH IgG1, κ |
| APC | anti-CD4 | RM4-5 | BD Pharmingen | Rat IgG2α, κ |
| APC | anti-TCRβ | H57-597 | Biolegend | ArH IgG |
| APC | anti-TCR-γδ | GL3 | eBioscience | ArH IgG |
| APC | anti-TCRVγ1 | 2.11 | Biolegend | ArH IgG |
| APC-cy7 | anti-CD4 | RM4-5 | BD Pharmingen | Rat IgG2α, κ |
| APC-CY7 | anti-IFN-γ | XMG1.2 | BD Pharmingen | Rat IgG1, κ |
| APC-cy7 | anti-NK1.1 | PK136 | Biolegend | Ms IgG2a, κ |

**Supplemental Table 2. The primers for each gene detected by real-time PCR**

| ***Gene*** | ***Forward primer (5’-3’)*** | ***Reverse primer (5’-3’)*** | ***Amplicon***  ***length (bp)*** | ***Ref*** |
| --- | --- | --- | --- | --- |
| ***RORc*** | gaacttggggaaccagaaca | ttggcaaactccaccacata | 290 | *This work* |
| ***SOX13*** | ctgagcagaagaacatggcc | tgcagggaatgggctgaata | 258 | *This work* |
| ***IL-17A*** | tccagaaggccctcagacta | agcatcttctcgaccctgaa | 239 | ([Miller *et al.* 2015](#_ENREF_8)) |
| ***IL-17F*** | agaagcagccattggagaaa | ggggtctcgagtgatgttgt | 289 | *This work* |
| ***ICOS*** | ttacttctgcagcctgtcca | tcatgcacactggatccgta | 210 | *This work* |
| ***CD44*** | caacgaagatgcctggtacg | ggttccggggtccaattttc | 307 | *This work* |
| ***IL-21*** | atgcagcttttgcctgtttt | gggaatcttctcggatcctc | 289 | *This work* |
| ***IL-1R1*** | caacgtgagcttcttcggag | cgtgacgttgcagatcagtt | 244 | ([Cheng *et al.* 2017](#_ENREF_2)) |
| ***BLK*** | cttgccccaccatctcctaa | tctctactggggccacaaag | 214 | *This work* |
| ***MAF*** | cccacacataccctggactt | ggaaacacagcaagctccaa | 261 | *This work* |
| ***Lingo4*** | tctctcctttggtcttggca | ggaggagcaggaaaaggagt | 288 | *This work* |
| ***CD163L1*** | cctggctgcacaatgtatcc | caaaacactgtggcctcctc | 244 | *This work* |
| ***SLAMF1*** | catcctggttttcacggcaa | ctggcataaactgtggtggg | 240 | *This work* |
| ***TOX2*** | caatgagccacagaagccag | cttggcagcttcagtcttcc | 181 | *This work* |
| ***BCL11b*** | cgatgccagaatagatgccg | agcgggaagttcatctgaca | 225 | *This work* |
| ***CCR6*** | GCCACTCTAATCAGTAAGACTTCA | TTGTCATAATCATCCGTTCCA | 180 | *This work* |
| ***CCR2*** | ggagccatacctgtaaatgcc | caggaagagcaggtcagaga | 436 | *This work* |
| ***DKK3*** | ggaggaagctacgctcaatg | ggcccacagtcttcatcaat | 339 | *This work* |
| ***IL-18R1*** | GGGCTACTACTCCTGCGTGTT | CCTCTTTCCTGATGCTCCAA | 224 | *This work* |
| ***TNF-α*** | ccacatctccctccagaaaa | agggtctgggccatagaact | 259 | ([Sartoretto *et al.* 2018](#_ENREF_9)) |
| ***CSF2*** | actacaatggcccacgagag | ccgtagaccctgctcgaata | 285 | *This work* |
| ***Itgae(CD103)*** | gaggacagagaggaggatgc | gtgtgtgtgccaaggagaag | 217 | *This work* |
| ***IL7R*** | agacagcccatctccacttc | tcctgcattcaccacatcct | 385 | *This work* |
| ***Il23R*** | AAAATCATCCCACGAACC | CTGGCATTATCATTCTCAAA | 297 | *This work* |
| ***IL-22*** | ccgaggagtcagtgctaagg | agcttcttctcgctcagacg | 314 | *This work* |
| ***β-actin*** | TGACGTTGACATCCGTAAAGACC | CTCAGGAGGAGCAATGATCTTGA | 148 | ([Mathew *et al.* 2005](#_ENREF_7)) |

**Supplemental Table 3. Clinical characteristics of NSCLC patients in this study**

|  | **Young** | **Aged** | **P value** |
| --- | --- | --- | --- |
| **Age**  **mean (min-max)** | 42.6 (20-50) | 77.5 (75-86) |  |
| **Sex (M/F)** | 20/8 | 22/10 | 0.821 |
| **Pathologic stage** |  |  | 0.503 |
| **I** | 10 | 16 |  |
| **II** | 9 | 9 |  |
| **III** | 9 | 7 |  |
| **Pathologic Typing** |  |  | 0.694 |
| Squ | 11 | 11 |  |
| Ade | 17 | 21 |  |
| **Total number** | 28 | 32 |  |

Pathologic stage was determined as I, II, III or IV according to the American Joint Committee On Cancer (AJCC) TNM system. Squ: squamous epithelial carcinoma; Ade: adenocarcinoma; NSCLC: no small cell lung cancer.

**Supplemental Figure legends**

**sFigure 1. Cytokine production of CD4**^+^**T and NKT cells in lung and spleen of aged mice compared with young mice.** The MNCs were isolated and analyzed using FACS. The CD4^+^T (CD3^+^ αβTCR^+^CD4^+^) and NKT (CD3^+^ NK1.1^+^) cells in the lungs (A) and spleen (B) were gated to analyze the frequency and number of IL-17A positive or IFN-γ positive cells respectively. There were six mice in each group. The data are shown as the mean ± SEM. Student’s *t-*test was used. *p<0.05, **p<0.01.

**sFigure 2. Cytokine production of CD4^+^T and NKT cells in lung and spleen of aged mice challenged with B16/F10 cells compared with young mice.** Young and aged mice were challenged with B16/F10 cells (1×10^5^ cells/mouse, i.v.). On day 21 after the B16/F10 challenge, the MNCs were isolated and analyzed by FACS. The CD4^+^T (CD3^+^ αβTCR^+^CD4^+^) and NKT (CD3^+^ NK1.1^+^) cells in the lungs (A) and spleen (B) were gated to analyze the frequency and number of IL-17A positive or IFN-γ positive cells respectively. There were six mice in each group. The data are shown as the mean ± SEM. Student’s *t-*test was used. *p<0.05, **p<0.01.

**sFigure 3. The number of lung-resident γδT cells did not alter in the co-cultured aged mice.** In the co-culture group, aged mice were co-housed with young mice for 4 weeks. (A) Body weight of the co-cultured aged mice were compared with the controls (n = 10/group). (B) Lung index of the co-cultured aged mice were compared with the controls (n = 6/group). Lung index = weight of lung (g)×10/body weight (g). (C) The total number of MNCs in the lungs (n=6). (D) The percentages of γδT (CD3^+^ γδTCR^+^) cells in the lymphocytes of the lungs were analyzed (n=6). (E) The numbers of γδT (CD3^+^ γδTCR^+^) cells in the lungs were analyzed (n=6). The data are shown as the mean ± SEM. Analysis of variance (one-way ANOVA) was used. *p<0.05.

**sFigure 4. No difference in the ability of B16/F10 tumor cells to colonize the lungs between the aged mice and the young mice.** Young and aged mice were challenged with GFP-transfected B16/F10 cells (1×10^5^ cells/mouse, i.v.). The tumor cells colonized in the lung tissue were detected as GFP positive cells by immunohistochemical staining analysis 48h after the GFP-transfected B16/F10 challenge. Arrows represent the B16/F10 tumor cells (brown) penetrating pulmonary blood vessels.

Supplemental Figure 1

Supplemental Figure 2

Supplemental Figure 3

Supplemental Figure 4
